# From one side to the other: what is essential? Perception of oncology patients and their caregivers in the beginning of oncology treatment and in palliative care

**DOI:** 10.1590/S1679-45082014RC3091

**Published:** 2014

**Authors:** Bruna Antenussi Munhoz, Henrique Soares Paiva, Beatrice Martinez Zugaib Abdalla, Guilherme Zaremba, Andressa Macedo Paiva Rodrigues, Mayra Ribeiro Carretti, Camila Ribeiro de Arruda Monteiro, Aline Zara, Jussara Oliveira Silva, Widner Baptista Assis, Luciana Campi Auresco, Leonardo Lopes Pereira, Adriana Braz del Giglio, Ana Claudia de Oliveira Lepori, Damila Cristina Trufelli, Auro del Giglio

**Affiliations:** 1 Faculdade de Medicina do ABC, Santo André, SP, Brazil.

**Keywords:** Neoplasm, Palliative care, Caregivers, Communication, Pacient acceptance of health care

## Abstract

**Objective:**

To evaluate the perception of oncology patients and their caregivers upon diagnosis and beginning of the therapy and during palliative care.

**Methods:**

A cross-sectional study at the oncology and palliative care outpatients clinics of the *Faculdade de Medicina do ABC* ***.*** Clinical and demographic data from patients and their caregivers were collected and questionnaires regarding the elements considered important in relation to the treatment were applied.

**Results:**

We enrolled 32 patients and 23 caregivers that were initiating treatment at the oncology outpatient clinic, as well as 20 patients and 20 caregivers at the palliative care clinic. Regarding the patients treated at the oncology clinic, the issues considered most important were a physician available to discuss the disease and answer questions (84%), trust in the physician (81%), and a physician with accessible language (81%). For their caregivers, the following issues were considered extremely important: trust in the medical team that treats the patients (96%), and the same medical team taking care of their relatives (87%). As to patients treated at the palliative care clinic, trust in the physician (83%), to be with people considered important to them (78%), and to be treated preserving their dignity (72%) were considered extremely important. For their caregivers, to receive adequate information about the disease and the treatment’s risks and benefits (84%), and sincere communication of information about the disease (79%) were considered extremely relevant.

**Conclusion:**

Confidence through good communication and consistency in care were fundamental values to achieve satisfaction among caregivers and patients with cancer during all the course of disease development.

## INTRODUCTION

Cancer is a universal disease with increasing numbers of patients affected; it is already the second cause of death in the United States^(1)^ and the third in Brazil,^(2)^ demonstrating its importance in terms of public health. With new diagnostic and therapeutic methods, it is possible to make early diagnosis, besides increasing survival, guaranteeing a greater number of people living with the disease. However, in detriment of the therapeutic advances and of the increased number of diagnoses in oncology, the mortality rates due to cancer continue to climb: in 2008, 7.6 million people died because of the disease, as per the World Health Organization.^(3)^

This panorama reflects the importance of implementing treatments and interventions for improved quality of life of all oncology patients and their caregivers, of cure whenever possible, and of the care of symptoms – when a cure is no longer an attainable goal.^(3)^ Currently, there is a lot of research regarding cancer, but most of it is based on the physician’s perspective, distanced from the doctor-patient relationship and of the benefits that can come when this is adequately established.^(4)^ Additionally, the perspective of the caregivers becomes significant since these patients are a part of a family dynamic, which is altered as of the diagnosis until resolution or the palliative phase, impacting the evolution of the disease. A Canadian study^(4)^ covered the perspective of patients and their caregivers regarding the most important elements related to oncological treatment they are given and the interaction with the healthcare team within the context of palliative care. In Brazil, there is little knowledge of what is really important to oncology patients and their caregivers, not only at the time of the diagnosis of cancer, when they still know little about the disease and have not yet been submitted to treatment, but also during palliative care.

## OBJECTIVE

To evaluate the perception of oncology patients and their caregivers at the beginning of their diagnostic and therapeutic approach, and during palliative care; to establish comparisons among patients and subgroups of caregivers in both circumstances, highlighting the convergent and divergent points as to what each group considers most important at the time.

## METHODS

After approval by the Institutional Review Board (Opinion number 224,218, dated March 20, 2013, CAAE number 13382013.0.0000.0082), patients seen at the outpatient clinics for symptom control (palliative care) and at the first clinical visit at the oncology outpatients clinics of the *Faculdade de Medicina do ABC do Hospital Estadual Mário Covas*, in Santo André, SP, and of the *Hospital de Ensino Padre Anchieta, *in São Bernardo do Campo, SP, were invited to participate during the period from April to June, 2013.

Patients over 18 years of age with confirmed diagnosis of neoplasm and/or with advanced disease were considered eligible for the study; excluded were those who had a significant cognitive deficit that impeded the application of the questionnaire, as well as the illiterate patients or those not fluent in portuguese. All those who agreed to participate in the study signed the informed consent form (ICF), making clear the voluntary characteristic of their participation. After the decision of the patient to participate in the study, the invitation was extended to the caregivers to also take part.

The companions were considered caregivers when they had some degree of bond with the patient, were involved in their treatment, or reported some form of contact with him/her at least once a week. They could be family members or not, as one patient could have more than one caregiver. The ICF was also delivered and signed by the caregivers, as long as they demonstrated the capacity to understand the study text and were aged over 18 years at the time when they were approached. Patients who had no caregivers were invited to participate in the study with no difference relative to those who had caregivers.

Clinical and demographic data were collected from the patients, along with demographic data from the caregivers. The clinical questionnaire covered information on the disease, such as type of neoplasm, staging, date of diagnosis, comorbidities, and past clinical/surgical and family history. The data in reference to the disease that patients were not able to inform was obtained from their medical records. As to demographic data, questions were asked about level of schooling, marital status, age, ethnicity, place of residence, place of birth, religion, and level of spirituality. The caregivers were also asked about their degree of kinship or bond with the patient, for those who were not relatives.

Next, the patients and their caregivers received questionnaires in reference to the topics to be included as significant for the treatment. These questionnaires were prepared based on a Canadian study^(4)^ applied to hospitalized palliative patients. In order for the questionnaires to be applied within the context of our study and of our services, some questions were changed and adapted.

Basically, the original questionnaire was developed based on a review of literature pertaining to palliative care and additional items, generated from a new review and from a multidisciplinary discussion held at Queen University, in Ontario. This was tested in interviews with a group of 12 severely ill patients, even though eligible for the study, so that the final 28 items were comprehensively tested among healthcare employees and patients and their caregivers in order to attain clarity, objectivity, and sensitivity.^(4)^

For the alterations to be made, the following diverging points were considered: the moment of application (the original questionnaire only focuses on palliative patients, so some items in reference of the final stages of life, such as the preferential location for death, were excluded from the questionnaire applied at the time subsequent to the diagnosis), the fact that the individuals were outpatients (excluding the item in reference to hospitalization), and the social economic differences between the two countries (since health care is systematized differently in the two places, such as in regard to the nursing services).

Thus, the questionnaires for patients and caregivers continued to cover the same five domains (Medical and Nursing care; communication and decision making; social and support relations; meaning of existence; and early planning for care). The questionnaire that was applied at the time right after the diagnosis contained 22 items for the patients and 17 for the caregivers; whereas the questionnaire applied for patients under palliative care, had 24 items for the patents and 21 for the caregivers.

Each one of these items was evaluated as to its importance for the patient or caregiver, using a scale from zero to 4, where zero meant no importance at all, 1 not very important, 2 somewhat important, 3 very important, and 4 extremely important. According to the score given for each item, we listed, in decreasing order, starting from the frequency of the items considered “extremely important”, and these were classified according to the proportion of the responses.

## RESULTS

During the period of April and May 2013, 32 patients and 23 caregivers who initiated treatment at the oncology outpatients clinics were enrolled. The mean age was 61 years (range: 26 to 84 years) and 43.5 years (range: 26 to 63 years), and the prevalence was of the female gender (56% and 83%), respectively. As to these caregivers and their degree of kinship, 43% of them were sons and daughters. For these patients, the items considered most important were that the physician be available to discuss their illness and answer their questions (84%), the importance of having confidence in the physicians who care for you (81%), and that the physician speak in a manner that you understand with accessible language (81%) (Table 1). For their caregivers, the following aspects were considered extremely important: having confidence in the physicians who care for the patient (96%) and always having the same medical team caring for your family member (87%) (Table 2).

**Table 1:** Answers of the patients during oncologic treatment

Item	0	1	2	3	4
	n (%)	n (%)	n (%)	n (%)	n (%)
Physician available to discuss about the disease and answer questions	0	0	1 (3)	4 (13)	27 (84)
Confidence in the physicians who care for you	0	0	0	6 (19)	26 (81)
A physician with accessible language	0	0	1 (3)	5 (16)	26 (81)
To receive adequate information on the disease, including the risks and benefits of the treatments	0	0	1 (3)	7 (22)	25 (78)
To know the physician responsible for your care	0	0	1 (3)	7 (22)	25 (78)
The disease be stated in an objective manner	1 (3)	0	0	6 (19)	25 (78)
To always have the same medical team caring for you	0	0	0	5 (16)	25 (78)
To receive care in a respectful way	0	0	0	8 (25)	24 (75)
To be involved in the decisions about your treatment and care	0	0	0	8 (25)	24 (75)
To have your spiritual and religious needs respected	0	0	1 (3)	8 (25)	23 (72)
To be treated in such a way that preserves your dignity	0	0	0	10 (31)	22 (69)
To have someone to accompany you when you are sad, anxious, or afraid	1 (3)	0	1 (3)	9 (28)	22 (69)
To be treated with respect for your needs, values, and preferences	0	0	1 (3)	9 (28)	22 (69)
To maintain a relationship or intensify your relationship with people who you consider important	0	0	0	11 (34)	21 (66)
To have a feeling of control over your decisions and your care	0	0	1 (3)	10 (31)	21 (66)
To receive help to make difficult decisions	0	0	0	10 (31)	21 (66)
Physician discuss concerns relative to your disease and your care with your family	0	0	1 (3)	12 (38)	19 (59)
To contribute with time, gifts, knowledge, and experience	0	1 (3)	0	12 (38)	19 (59)
Opportunity to discuss your fears regarding the disease and its course	1 (3)	0	3 (9)	9 (28)	19 (59)
That the disease does not create financial problems in your family	1 (3)	0	1 (3)	13 (41)	17 (53)
To have relief of symptoms	1 (3)	0	2 (6)	12 (38)	17 (53)
To not feel like an emotional or physical burden on your family	3 (9)	1 (3)	3 (9)	12 (38)	13 (41)

4: extremely important; 3: very important; 2: somewhat important; 1: not very important; 0: no importance.

**Table 2:** Responses of the caregivers of patients in oncologic treatment

Item	0	1	2	3	4
	n (%)	n (%)	n (%)	n (%)	n (%)
To have confidence in the physicians who care for the patient	0	0	0	1 (4)	22 (96)
To always have the same medical team caring for your family member	0	0	1 (4)	2 (9)	20 (87)
Communication about the disease should be stated in an objective manner	0	0	0	5 (22)	18 (78)
To know which physician is responsible for your relative’s care	0	0	0	5 (22)	18 (78)
The physician is available to discuss about the diseases and answer your questions	0	0	0	8 (35)	16 (70)
Patient has relief of symptoms	0	0	0	7 (30)	16 (70)
To be involved in the decisions as to treatment and care	0	0	0	9 (39)	14 (61)
To receive care in a respectful manner	0	0	0	9 (39)	14 (61)
To have a physician who speaks in a manner you can understand, with accessible language	0	0	0	9 (39)	14 (61)
To have a physician who discusses concerns related to the diseases and its care	0	1 (4)	0	8 (35)	14 (61)
Feeling of control over the decisions and care	0	0	0	10 (43)	13 (57)
To maintain a relationship or intensify your relationship with the people you consider important	0	0	1 (4)	9 (39)	13 (57)
To receive help in making difficult decisions	0	0	0	11 (48)	12 (52)
That the disease does not create financial problems	1 (4)	0	2 (9)	9 (39)	12 (52)
To have your spiritual and religious needs respected	0	0	2 (9)	9 (39)	12 (52)
Contribute with time, gifts, knowledge, and experience with others	0	0	2 (9)	10 (43)	11 (48)
To have someone to accompany you when you are sad, anxious, confused, or afraid	0	0	0	13 (57)	10 (43)

4: extremely important; 3: very important; 2: somewhat important; 1: not very important; 0: no importance.

During the same period, 20 patients and 20 caregivers were included in the symptom-control outpatient clinics, with a mean age of 69 (range: 23 to 84) and 47 (range: 20 to 62) years, respectively. Female prevalence in both groups was 75% and as to degree of kinship between caregiver and patient, 65% of them were sons or daughters. For the patients, the following items were considered extremely important: to have confidence in the physicians that care for you (83%), to be able to have people near who are important to you (78%), and to be treated in a manner that preserves your dignity (72%) (Table 3). For the caregivers, the elements considered extremely important were to receive adequate information on their relative’s disease, the risks and benefits of treatment (84%), and that the information regarding your loved one’s illness be communicated in a sincere manner (79%) (Table 4).

**Table 3:** Responses of patients under palliative care

Item	0	1	2	3	4
	n (%)	n (%)	n (%)	n (%)	n (%)
To have confidence in the physicians who care for you	0	0	0	6 (33)	15 (83)
To be with people who are important to you	0	0	0	6 (33)	14 (78)
To be treated with dignity	0	0	4 (22)	7 (39)	13 (72)
To receive respectful and humanized treatment	0	0	1 (6)	7 (39)	12 (67)
To be treated as an individual and not as a disease	0	0	0	8 (44)	12 (67)
To have relief of symptoms	0	0	0	7 (39)	12 (67)
To receive help in decisions about treatment	0	1 (6)	0	8 (44)	11 (61)
That information on the disease be communicated in a sincere manner	0	0	2 (11)	6 (33)	12 (67)
To have adequate information on the disease and the risks and benefits of treatments	0	0	1 (6)	8 (44)	11 (61)
To have someone present when you are feeling sad, fearful, or anxious	0	1 (6)	1 (6)	7 (39)	11 (61)
To be able to contribute towards others	0	2 (11)	0	8 (44)	10 (56)
To have a physician available to talk about the disease and answer questions	0	0	1 (6)	3 (17)	10 (56)
To have a physician who talks to your family about the disease and treatment	1 (6)	0	0	9 (50)	11 (61)
That the disease does not cause financial problems for your family	2 (11)	1 (6)	4 (22)	3 (17)	11 (61)
That the physician speaks in a manner that you understand	0	0	0	11 (61)	11 (61)
To have the feeling of control over your decisions	0	2 (11)	0	10 (56)	10 (56)
To complete tasks and prepare for final stages of your life	1 (6)	0	1 (6)	9 (50)	11 (61)
To have your religious and spiritual needs met	1 (6)	1 (6)	1 (6)	8 (44)	11 (61)
To not be a physical and emotional burden for your family	2 (11)	6 (33)	0	5 (28)	10 (56)
To know which physician is responsible for you	1 (6)	0	1 (6)	11 (61)	11 (61)
To be involved in the decisions regarding your treatment	1 (6)	2 (11)	3 (17)	8 (44)	10 (56)
To not be kept alive by means of machines when there is no hope of a significant recovery	3 (17)	4 (22)	2 (11)	6 (33)	10 (56)
To have the opportunity to discuss your fears regarding death	8 (44)	2 (11)	1 (6)	2 (11)	11 (61)
To be able to die in the location of your preference	0	4 (22)	2 (11)	10 (56)	10 (56)

4: extremamente importante; 3: muito importante; 2: pouco importante; 1: não muito importante; 0: sem importância.

**Table 4:** Responses of caregivers of patients under palliative care

Item	0	1	2	3	4
	n (%)	n (%)	n (%)	n (%)	n (%)
To receive adequate information on your loved one’s illness and on the risks and benefits of the treatment	0	0	0	5 (26)	16 (84)
Information regarding the disease is communicated in a sincere manner	0	0	0	6 (32)	15 (79)
To have a physician who explains about the disease in a way you can understand	0	0	0	6 (32)	15 (79)
That the family member receives relief of symptoms	0	0	1 (5)	5 (26)	15 (79)
To receive respectful and humanized treatment	0	0	0	7 (37)	14 (74)
To have a feeling of control over decisions regarding treatment	0	0	2 (11)	8 (42)	11 (58)
To have confidence in the physicians	0	0	0	10 (53)	11 (58)
To complete tasks and resolve conflicts	0	0	0	10 (53)	11 (58)
To receive help in making difficult decisions	0	0	0	10 (53)	11 (58)
To have someone to talk to when you are feeling sad, anxious, or fearful	0	0	0	11 (58)	10 (53)
To have the opportunity to maintain and increase the relationship with the patient	0	0	1 (5)	9 (47)	11 (58)
To know which physician is responsible	0	0	1 (5)	8 (42)	12 (63)
To be involved in the decisions about treatment and care that the patient will receive	0	0	1 (5)	9 (47)	11 (58)
To have your religious and spiritual needs met	1 (5)	0	2 (11)	6 (32)	12 (63)
To have a physician who will talk with you about concerns regarding the disease	2 (11)	1 (5)	0	9 (47)	9 (47)
To be able to contribute towards others	0	0	0	12 (63)	9 (47)
To have a physician available to discuss about the disease and answer questions	0	0	0	12 (63)	9 (47)
That it be possible for your relative to die in a location of his/her preference	2 (11)	1 (5)	3 (16)	10 (53)	5 (26)
To have the opportunity to discuss about fear of the relative dying	1 (5)	1 (5)	3 (16)	6 (32)	4 (2%)
That the disease does not cause financial problems	8 (42)	1 (5)	4 (21)	4 (21)	4 (21)
To not have your relative maintained alive by means of machines when there is no hope for a satisfactory recovery	5 (26)	3 (16)	3 (16)	7 (37)	1 (5)

4: extremely important; 3: very important; 2: somewhat important; 1: not very important; 0: no importance.

## DISCUSSION

According to Chin et al.,^(5)^ the objective of medicine as a profession dedicated to cure and care for the patient in a dignified manner depends on a doctor-patient relationship sustained by trust. For this trust-based doctor-patient relationship to be built, communication must be made in an effective manner, with appropriate attention and language.

By means of this study, we were able to determine that the most important elements for the patients, when initiating their treatment, were the availability of the physician to discuss about the disease and answer questions, trust in the physician, and accessible and comprehensible language. The three elements, together, lead to the rhetoric importance of communication between the physician and the patient, giving rise to a relationship of confidence, that conveys credibility, knowledge, and includes donating time.^(3)^ To know how to speak, express oneself with clarity, and be able to communicate the information in the most didactic way possible enables fulfillment of the Hippocratic ideal of the Physician who cares for and can influence his/her patient.^(6)^ In fact, the origin of the word “doctor” is Latin: “*d’ocere*”, which means to teach.^(7)^ For this, a daily practice of medicine is necessary, having as fundamental pillar, the patient.^(8)^

According to the caregivers of patients initiating cancer treatment, the most important issues were confidence in the physicians who care for the patient and always having the same medical team caring for the family member. Confidence proves important not only in the relationship with the patient, but also for those inserted into the same context. The second most important element is interesting, since amidst the care given to a certain patient by the same professional or team over time, there is individualization of his/her care.^(9)^ This item is listed as relevant not only in our study,^(10^,^11)^ and in fact, there is a literature review on the satisfaction of patients during their treatment that advocates that the more personalized the care, the better the rates of satisfaction.^(9)^ According to this same study, individualization of treatment makes communication of the patient with the physician better, as well as the involvement of the patient with his/her treatment.^(9)^

As for the patients at our palliative care service, the most important item was having confidence in the physician responsible for the treatment, a paramount item for the patients in the Canadian study as well.^(4)^ In a study conducted by Thom et al.,^(12)^ medical behavior was correlated with patient confidence. These authors interviewed 414 patients who, after clinical visits, would evaluate their physicians as to 18 preestablished behaviors, which allowed an assessment of the degree of trust in these professionals. As a result, the behaviors most often associated with confidence in the physician were demonstration of attention and care, technical competence, and the quality of communication.

As to the caregivers of patients under palliative care, the three most important items mentioned in our study refer to quality of communication. In the Canadian study, such a concern of caregivers appeared in third place, and was also a priority.^(4)^ Nevertheless, to communicate in an adequate form may not be an easy task. A study by Wilmot and Hocker^(13)^ about physicians and healthcare professionals that cared for patients with advanced stage cancer in an American center showed that the most difficult part of their work was communication with the families of the patients. In a systematic review in which 21 papers were included, there was a positive correlation between effectiveness of communication between the physician and patient and the success of the treatment. The aspects that obtained the greatest relationships with adequate communication were pain control, compliance with the treatment, symptom resolution, and emotional health.^(14)^

In this way, in order to improve the quality of life of the patients and their caregivers, we need to know what is significantly important for both groups. We note that for patients, having trust in one’s physician, being received in a dignified and humanized manner, and keeping well informed as to one’s illness are priority issues. On the other hand, for the caregivers, most of the items judged as essential reflected the quality of communication with the physicians responsible for the care of their relatives, and that they have adequate control of the symptoms. Along the course of a chronic and potentially lethal disease, such as cancer, we have the opportunity to perceive which values remain important throughout time. Comparing these two studies at different time points of the natural progression of cancer at a given health center, the importance of trust in the healthcare team was clear, both as to diagnosis and in the more advanced phases of the disease. Knowledge of these factors allows a better focus of our efforts on what is, in fact, most important to patients and caregivers, and these same items can also serve as a focus for continuous evaluation, in order to detect our deficiencies and progresses in the same areas.^(15)^

## CONCLUSION

Confidence mediated by good communication and consistency in care are values considered fundamental for the satisfaction of caregivers and patients with cancer along the whole evolution of the illness. Structuring of services to enable longitudinal follow-up of patients and the cultivation of communication skills in medical training are probably the most promising attitudes needed to assure satisfaction of the population of patients with cancer and their caregivers.
