# Supplementary material for: From one side to the other: what is essential? Perception of oncology patients and their caregivers in the beginning of oncology treatment and in palliative care
Source: Einstein (Sao Paulo). 2014 Oct-Dec;12(4):485–91. doi: 10.1590/S1679-45082014RC3091 (PMC4879917; doi:10.1590/S1679-45082014RC3091)
Supplement: Translation

# Translation: De um lado ao outro: o que é essencial? Percepção dos pacientes oncológicos e de seus cuidadores ao iniciar o tratamento oncológico e em cuidados paliativos

**Authors:** Bruna Antenussi Munhoz, Henrique Soares Paiva, Beatrice Martinez Zugaib Abdalla, Guilherme Zaremba, Andressa Macedo Paiva Rodrigues, Mayra Ribeiro Carretti, Camila Ribeiro de Arruda Monteiro, Aline Zara, Jussara Oliveira Silva, Widner Baptista Assis, Luciana Campi Auresco, Leonardo Lopes Pereira, Adriana Braz del Giglio, Ana Claudia de Oliveira Lepori, Damila Cristina Trufelli, Auro del Giglio

**Affiliations:** 1 Faculdade de Medicina do ABC, Santo André, SP, Brasil.

**Keywords:** Neoplasias, Cuidados paliativos, Cuidadores, Comunicação, Aceitação pelo paciente de cuidados de saúde

## Abstract

**Objetivo:**

Avaliar a percepção dos pacientes oncológicos e de seus cuidadores no início da abordagem diagnóstica e terapêutica, e durante os cuidados paliativos.

**Métodos:**

Estudo transversal, realizado nos ambulatórios de oncologia e cuidados paliativos vinculados a Faculdade de Medicina do ABC. Foram coletados dados clínicos e demográficos de pacientes e de seus cuidadores, e aplicados questionários referentes aos elementos a serem elencados como importantes em relação ao tratamento.

**Resultados:**

Foram incluídos 32 pacientes e 23 cuidadores que iniciavam o tratamento nos ambulatórios de oncologia, bem como 20 pacientes e 20 cuidadores nos ambulatórios de cuidados paliativos. Em relação aos pacientes do ambulatório de oncologia, os itens considerados mais importantes foram: médico disponível para discutir a doença e responder dúvidas (84%), confiança nos médicos (81%) e médico com linguagem acessível (81%). Para seus cuidadores, os seguintes aspectos foram considerados extremamente importantes: confiança nos médicos que cuidam do paciente (96%) e mesma equipe médica cuidando do seu familiar (87%). Para pacientes do ambulatório de cuidados paliativos, foram considerados extremamente importantes: confiança nos médicos (83%), estar com pessoas consideradas importantes (78%) e ser tratado de modo que preservasse sua dignidade (72%). Para seus cuidadores, foram considerados extremamente importantes: receber informação adequada sobre a doença e os riscos e benefícios do tratamento (84%), e sinceridade na comunicação das informações a respeito da doença (79%).

**Conclusão:**

Confiança mediada por uma boa comunicação e a constância do cuidado foram valores tidos como fundamentais para a satisfação de cuidadores e de pacientes com câncer durante toda a evolução da doença.

## INTRODUÇÃO

O câncer é uma doença universal com números crescentes de pessoas acometidas, de modo que já corresponde à segunda causa de morte nos Estados Unidos^(1)^ e à terceira no Brasil,^(2)^ demonstrando sua importância em termos de saúde pública. Com os novos métodos diagnósticos e terapêuticos, há a possibilidade de um diagnóstico precoce, além de aumento da sobrevida, garantindo um maior número de pessoas convivendo com a doença. No entanto, em detrimento dos avanços terapêuticos e do aumento do número de diagnósticos na oncologia, os índices de mortalidade por câncer continuam aumentando: em 2008, 7,6 milhões de pessoas morreram por causa da doença, segundo a Organização Mundial da Saúde.^(3)^

Esse panorama reflete a importância de implementar terapêuticas e intervenções em prol da melhor qualidade de vida de todos os pacientes oncológicos e de seus cuidadores, da cura quando possível e do cuidado dos sintomas − quando a cura deixa de ser uma meta exequível.^(3)^ Atualmente, há muitas pesquisas a respeito do câncer, porém a maioria é baseada na perspectiva do médico, distanciando-se da relação médico-paciente e dos benefícios que podem advir quando esta é estabelecida de forma adequada.^(4)^ Além disso, a perspectiva de seus cuidadores se torna importante, pelo fato de que esses pacientes estão inseridos numa dinâmica familiar, que é alterada a partir do diagnóstico, até a resolução ou a fase paliativa, impactando na evolução de sua doença. Estudo canadense^(4)^ abordou a perspectiva dos pacientes e de seus cuidadores acerca de quais são os elementos mais importantes em relação ao tratamento oncológico que lhes é ministrado e da interação com a equipe de saúde, no contexto de cuidados paliativos. No Brasil, há pouco conhecimento do que realmente importa para os pacientes oncológicos e cuidadores, não só no momento do diagnóstico do câncer, quando ainda desconhecem a doença e não foram submetidos ao seu tratamento, como também durante os cuidados paliativos.

## OBJETIVO

Avaliar a percepção dos pacientes oncológicos e de seus cuidadores no início de sua abordagem diagnóstica e terapêutica, e durante os cuidados paliativos; estabelecer comparações entre os pacientes e os subgrupos de cuidadores nas duas circunstâncias, evidenciando os pontos convergentes e discrepantes, em relação ao que cada grupo considera mais importante em cada momento.

## MÉTODOS

Após aprovação pelo Comitê de Ética em Pesquisa da instituição (parecer número 224.218, de 20 de março de 2013, CAAE 13382013.0.0000.0082), foram convidados a participar do estudo os pacientes atendidos nos ambulatórios de controle de sintomas (cuidados paliativos) e em primeira consulta nos ambulatórios de oncologia da Faculdade de Medicina do ABC do Hospital Estadual Mário Covas, em Santo André, SP, e do Hospital de Ensino Padre Anchieta, em São Bernardo do Campo, SP, no período de abril a junho de 2013.

Foram considerados elegíveis ao estudo os pacientes maiores de 18 anos com diagnóstico confirmado de neoplasia e/ou com doença avançada; foram desconsiderados aqueles que tinham algum défice cognitivo importante que impedisse a aplicação do questionário, bem como os pacientes analfabetos ou não fluentes no português. Todos que concordaram em participar do estudo assinaram o Termo de Consentimento Livre Esclarecido (TCLE), deixando claro o caráter voluntário da participação. Após a decisão do paciente em participar do estudo, estendia-se o convite para que o cuidador também participasse.

Os acompanhantes foram considerados cuidadores quando tivessem algum vínculo com o paciente, estivessem envolvidos em seu tratamento ou relatassem algum contato com ele pelo menos uma vez por semana. Eles podiam ser ou não familiares do paciente, sendo que um paciente poderia ter mais de um cuidador. O TCLE também foi entregue e assinado pelos cuidadores, desde que demonstrassem aptidão ao entendimento do texto do estudo e que fossem maiores de 18 anos no ato da abordagem. Os pacientes que não tinham cuidadores foram convidados a participar do estudo sem qualquer diferença em relação àqueles com cuidadores.

Foram coletados dados clínicos e demográficos dos pacientes, bem como dados demográficos dos cuidadores. O questionário clínico versou sobre dados da doença, como tipo de neoplasia, estadiamento, data do diagnóstico, comorbidades, antecedentes pessoais e familiares. Os dados referentes à doença que o paciente não soube informar foram obtidos do prontuário do mesmo. Quanto aos dados demográficos, eram interrogados: escolaridade, estado civil, idade, etnia, procedência, naturalidade, religião e o nível de espiritualidade. Aos cuidadores, foi ainda perguntado qual era o grau de parentesco com o paciente ou seu vínculo, para aqueles que não eram familiares.

Em seguida, foram entregues para os pacientes e seus cuidadores os questionários referentes aos elementos a serem elencados como importantes em relação ao tratamento. Esses questionários foram elaborados com base em um trabalho canadense^(4)^ aplicado em pacientes paliativos internados. Para que os questionários pudessem ser aplicados no contexto do nosso estudo e de nossos serviços, algumas questões foram alteradas e adaptadas.

Basicamente, o questionário original foi desenvolvido a partir de uma revisão da literatura em cuidados paliativos e itens adicionais, gerados a partir de nova revisão e de uma discussão multidisciplinar, realizada na Universidade Queen, em Ontário. Este foi testado em entrevistas com um grupo de 12 pacientes graves, porém elegíveis ao estudo, para que os 28 itens finais fossem, então, testados de maneira abrangente, entre funcionários da saúde e pacientes e seus cuidadores, de modo a atingir clareza, objetividade e sensibilidade.^(4)^

Para as alterações serem feitas, foram considerados os seguintes pontos divergentes: o momento de aplicação (o questionário original só aborda pacientes paliativos, logo alguns itens referentes ao fim da vida, como a escolha preferencial para o local do óbito, foram excluídos do questionário aplicado no momento subsequente ao diagnóstico), o fato dos pacientes serem ambulatoriais (excluindo o item referente à internação hospitalar) e as diferenças socioeconômicas entre os dois países (já que a sistematização da saúde é diferente nos dois lugares, como, por exemplo, em relação ao serviço da Enfermagem).

Assim, os questionários para pacientes e cuidadores continuaram a abordar os mesmos cinco domínios (cuidados médicos e de Enfermagem; comunicação e tomada de decisão; relações sociais e de apoio; significado da existência; e planejamento antecipado de atendimento). O questionário que foi aplicado no momento subsequente ao diagnóstico continha 22 itens para os pacientes e 17 para os cuidadores; já o questionário aplicado para pacientes em cuidados paliativos, 24 itens para os pacientes e 21 para os cuidadores.

Cada um destes itens foi avaliado quanto à sua importância para o paciente ou cuidador, em uma escala de zero a 4, sendo zero sem importância alguma, 1 não muito importante, 2 um pouco importante, 3 muito importante e 4 extremamente importante. De acordo com a pontuação dada para cada item, elencamos, em ordem decrescente, a partir da frequência dos itens considerados “extremamente importante”, e estes foram classificados de acordo com a proporção das respostas.

## RESULTADOS

No período de abril e maio de 2013, foram incluídos 32 pacientes e 23 cuidadores que iniciavam o tratamento nos ambulatórios de oncologia. A média de idade foi de 61 (variação de 26 a 84) e 43,5 (variação de 26 a 63) anos, e a prevalência foi do sexo feminino (56% e 83%), respectivamente. Em relação a esses cuidadores e ao seu grau de parentesco, 43% deles eram filhos. Para esses pacientes, os itens considerados mais importantes foram: que o médico esteja disponível para discutir sua doença e responder suas dúvidas (84%), a importância em ter confiança nos médicos que cuidam de você (81%) e que o médico fale de uma maneira que você compreenda com linguagem acessível (81%) (Tabela 1). Para seus cuidadores, os seguintes aspectos foram considerados extremamente importantes: a importância em ter confiança nos médicos que cuidam do paciente (96%) e ter sempre a mesma equipe médica cuidando de seu familiar (87%) (Tabela 2).

**Tabela 1:** Respostas dos pacientes durante o tratamento oncológico

Item	0	1	2	3	4
	n (%)	n (%)	n (%)	n (%)	n (%)
Médico disponível para discutir a doença e responder dúvidas	0	0	1 (3)	4 (13)	27 (84)
Confiança nos médicos que cuidam de você	0	0	0	6 (19)	26 (81)
Médico com linguagem acessível	0	0	1 (3)	5 (16)	26 (81)
Receber informações adequadas sobre a doença, incluindo riscos e benefícios dos tratamentos	0	0	1 (3)	7 (22)	25 (78)
Conhecer o médico responsável pelo seu cuidado	0	0	1 (3)	7 (22)	25 (78)
Doença ser dita de maneira objetiva	1 (3)	0	0	6 (19)	25 (78)
Ter sempre a mesma equipe médica cuidando de você	0	0	0	5 (16)	25 (78)
Receber cuidados de maneira respeitosa	0	0	0	8 (25)	24 (75)
Estar envolvido nas decisões sobre seu tratamento e cuidados	0	0	0	8 (25)	24 (75)
Ter suas necessidades espirituais e religiosas respeitadas	0	0	1 (3)	8 (25)	23 (72)
Ser tratado de uma maneira que preserva sua dignidade	0	0	0	10 (31)	22 (69)
Ter alguém que o acompanhe quando estiver triste, ansioso ou com medo	1 (3)	0	1 (3)	9 (28)	22 (69)
Ser tratado com respeito a suas necessidades, valores e preferências	0	0	1 (3)	9 (28)	22 (69)
Manter uma relação ou intensificar seu relacionamento com pessoas que você considera importante	0	0	0	11 (34)	21 (66)
Sentimento de controle sobre suas decisões e seus cuidados	0	0	1 (3)	10 (31)	21 (66)
Receber ajuda para tomar decisões difíceis	0	0	0	10 (31)	21 (66)
Médico discute preocupações em relação à sua doença e aos seus cuidados, junto de sua família	0	0	1 (3)	12 (38)	19 (59)
Contribuir com tempo, presentes, conhecimento e experiência	0	1 (3)	0	12 (38)	19 (59)
Oportunidade de discutir seus medos em relação à doença e ao seu percurso	1 (3)	0	3 (9)	9 (28)	19 (59)
Doença não crie problemas financeiros na sua família	1 (3)	0	1 (3)	13 (41)	17 (53)
Ter alívio de sintomas	1 (3)	0	2 (6)	12 (38)	17 (53)
Não se sentir um peso emocional ou físico para sua família	3 (9)	1 (3)	3 (9)	12 (38)	13 (41)

4: extremamente importante; 3: muito importante; 2: pouco importante; 1: não muito importante; 0: sem importância.

**Tabela 2:** Respostas dos cuidadores de pacientes em tratamento oncológico

Item	0	1	2	3	4
	n (%)	n (%)	n (%)	n (%)	n (%)
Ter confiança nos médicos que cuidam do paciente	0	0	0	1 (4)	22 (96)
Ter sempre a mesma equipe médica cuidando do seu familiar	0	0	1 (4)	2 (9)	20 (87)
Comunicação sobre a doença ser dita de maneira objetiva	0	0	0	5 (22)	18 (78)
Conhecer qual o médico responsável pelo cuidado com seu familiar	0	0	0	5 (22)	18 (78)
Médico disponível para discutir a doença e responder suas dúvidas	0	0	0	8 (35)	16 (70)
Ter alívio de sintomas do paciente	0	0	0	7 (30)	16 (70)
Estar envolvido nas decisões sobre tratamento e cuidados	0	0	0	9 (39)	14 (61)
Receber cuidados de maneira respeitosa	0	0	0	9 (39)	14 (61)
Médico que fale de uma maneira que você compreenda com linguagem acessível	0	0	0	9 (39)	14 (61)
Médico que discuta preocupações em relação à doença e seus cuidados	0	1 (4)	0	8 (35)	14 (61)
Sentimento de controle sobre as decisões e os cuidados	0	0	0	10 (43)	13 (57)
Manter uma relação ou intensificar seu relacionamento com as pessoas que considera importantes	0	0	1 (4)	9 (39)	13 (57)
Receber ajuda para tomar decisões difíceis	0	0	0	11 (48)	12 (52)
A doença não crie problemas financeiros	1 (4)	0	2 (9)	9 (39)	12 (52)
Ter suas necessidades espirituais e religiosas respeitadas	0	0	2 (9)	9 (39)	12 (52)
Contribuir com tempo, presentes, conhecimento e experiência com outros	0	0	2 (9)	10 (43)	11 (48)
Alguém que o acompanhe quando estiver triste, ansioso, confuso ou com medo	0	0	0	13 (57)	10 (43)

4: extremamente importante; 3: muito importante; 2: pouco importante; 1: não muito importante; 0: sem importância.

Durante o mesmo período, foram incluídos 20 pacientes e 20 cuidadores nos ambulatórios de controle de sintomas, sendo a média de idade de 69 (variação de 23 de 84) e de 47 (variação de 20 a 62) anos, respectivamente. A prevalência feminina em ambos os grupos foi de 75% e, em relação ao grau de parentesco do cuidador com o paciente, 65% deles eram filhos. Para os pacientes, foram considerados extremamente importantes: ter confiança nos médicos que cuidam de você (83%), poder estar com as pessoas que são importantes para você (78%) e ser tratado de modo que preserve sua dignidade (72%) (Tabela 3). Para os cuidadores, os elementos considerados extremamente importantes foram: receber informação adequada sobre a doença de seu parente e sobre os riscos e benefícios do tratamento (84%) e que as informações a respeito da doença do parente sejam comunicadas de maneira sincera (79%) (Tabela 4).

**Tabela 3:** Respostas dos pacientes em cuidados paliativos

Item	0	1	2	3	4
	n (%)	n (%)	n (%)	n (%)	n (%)
Confiança nos médicos que cuidam de você	0	0	0	6 (33)	15 (83)
Estar com pessoas importantes para você	0	0	0	6 (33)	14 (78)
Ser tratado com dignidade	0	0	4 (22)	7 (39)	13 (72)
Receber tratamento respeitoso e humanizado	0	0	1 (6)	7 (39)	12 (67)
Ser tratado como um indivíduo e não como uma doença	0	0	0	8 (44)	12 (67)
Ter alívio dos sintomas	0	0	0	7 (39)	12 (67)
Receber ajuda para decisões sobre o tratamento	0	1 (6)	0	8 (44)	11 (61)
Informações sobre a doença comunicadas de forma sincera	0	0	2 (11)	6 (33)	12 (67)
Informações adequadas sobre a doença e os riscos e benefícios dos tratamentos	0	0	1 (6)	8 (44)	11 (61)
Ter alguém presente quando estiver se sentindo triste, com medo ou ansioso	0	1 (6)	1 (6)	7 (39)	11 (61)
Ser capaz de contribuir com os outros	0	2 (11)	0	8 (44)	10 (56)
Médico disponível para conversar sobre a doença e tirar dúvidas	0	0	1 (6)	3 (17)	10 (56)
Médico que converse com sua família sobre a doença e o tratamento	1 (6)	0	0	9 (50)	11 (61)
Doença não traga problemas financeiros para sua família	2 (11)	1 (6)	4 (22)	3 (17)	11 (61)
Médico fale de um jeito que você entenda	0	0	0	11 (61)	11 (61)
Ter a sensação de controle sobre suas decisões	0	2 (11)	0	10 (56)	10 (56)
Completar tarefas e se preparar para o final da vida	1 (6)	0	1 (6)	9 (50)	11 (61)
Ter suas necessidades religiosas e espirituais realizadas	1 (6)	1 (6)	1 (6)	8 (44)	11 (61)
Não ser um peso físico e emocional para sua família	2 (11)	6 (33)	0	5 (28)	10 (56)
Saber qual o médico responsável por você	1 (6)	0	1 (6)	11 (61)	11 (61)
Estar envolvido nas decisões a respeito do seu tratamento	1 (6)	2 (11)	3 (17)	8 (44)	10 (56)
Não ser mantido vivo por meio de máquinas quando não houver esperança de uma recuperação significativa	3 (17)	4 (22)	2 (11)	6 (33)	10 (56)
Ter a oportunidade de discutir seus medos da morte	8 (44)	2 (11)	1 (6)	2 (11)	11 (61)
Poder morrer no lugar de sua preferência	0	4 (22)	2 (11)	10 (56)	10 (56)

4: extremamente importante; 3: muito importante; 2: pouco importante; 1: não muito importante; 0: sem importância.

**Tabela 4:** Respostas dos cuidadores dos pacientes em cuidados paliativos

Item	0	1	2	3	4
	n (%)	n (%)	n (%)	n (%)	n (%)
Receber informação adequada sobre a doença do seu parente e sobre os riscos e benefícios do tratamento	0	0	0	5 (26)	16 (84)
Informações a respeito da doença comunicadas de maneira sincera	0	0	0	6 (32)	15 (79)
Médico que explique sobre a doença de modo que você entenda	0	0	0	6 (32)	15 (79)
Parente tenha alívio dos sintomas	0	0	1 (5)	5 (26)	15 (79)
Tratamento respeitoso e humanizado	0	0	0	7 (37)	14 (74)
Sensação de controle sobre as decisões do tratamento	0	0	2 (11)	8 (42)	11 (58)
Confiança nos médicos	0	0	0	10 (53)	11 (58)
Completar tarefas e resolver conflitos	0	0	0	10 (53)	11 (58)
Receber ajuda para tomar decisões difíceis	0	0	0	10 (53)	11 (58)
Ter alguém para conversar quando estiver se sentindo triste, ansioso ou com medo	0	0	0	11 (58)	10 (53)
Oportunidade de manter e aumentar o relacionamento com o paciente	0	0	1 (5)	9 (47)	11 (58)
Saber qual o médico responsável	0	0	1 (5)	8 (42)	12 (63)
Estar envolvido nas decisões sobre o tratamento e cuidados que o paciente receberá	0	0	1 (5)	9 (47)	11 (58)
Ter suas necessidades religiosas e espirituais realizadas	1 (5)	0	2 (11)	6 (32)	12 (63)
Médico que converse sobre as preocupações da doença em conjunto	2 (11)	1 (5)	0	9 (47)	9 (47)
Ser capaz de contribuir com os outros	0	0	0	12 (63)	9 (47)
Médico disponível para discutir sobre a doença e responder dúvidas	0	0	0	12 (63)	9 (47)
Ser possível que seu parente morra em local da preferência dele	2 (11)	1 (5)	3 (16)	10 (53)	5 (26)
Ter a oportunidade de discutir sobre o medo do parente morrer	1 (5)	1 (5)	3 (16)	6 (32)	4 (2%)
Doença não traga problemas financeiros	8 (42)	1 (5)	4 (21)	4 (21)	4 (21)
Não ter seu parente mantido vivo por meio de aparelhos quando não houver esperanças de uma recuperação satisfatória	5 (26)	3 (16)	3 (16)	7 (37)	1 (5)

4:extremamente importante; 3: muito importante; 2: pouco importante; 1:não muito importante; 0: sem importância.

## DISCUSSÃO

Segundo Chin et al.,^(5)^ “os objetivos da medicina sendo esta uma profissão dedicada a curar e ao cuidado com o doente de uma forma digna dependem de uma relação médico paciente sustentada na confiança”. Para que haja a construção dessa relação médico-paciente embasada em confiança, é preciso que a comunicação seja feita de maneira efetiva, com atenção e linguagem adequadas.

Por meio desse estudo, pudemos constatar que os elementos mais importantes para os pacientes, ao iniciarem seu tratamento, foram: a disponibilidade do médico para discutir sua doença e responder suas dúvidas, confiança no médico e linguagem acessível e compreensível. Os três elementos, juntos, levam a retórica importância da comunicação entre o médico e seu paciente, fazendo surgir uma relação de confiança, que passe credibilidade, conhecimento e inclua doação de tempo.^(3)^ Saber falar, exprimir-se com clareza e conseguir veicular a informação da maneira mais didática possível nos remete ao ideal hipocrático do Doutor que cuida e que consegue influenciar seu paciente.^(6)^ De fato, a origem da palavra doutor é latina: “*d’ocere*”, que significa ensinar.^(7)^ Para tal, é necessária a prática diária de uma medicina, cujo pilar fundamental seja o paciente.^(8)^

Segundo os cuidadores dos pacientes que iniciavam o tratamento oncológico, as questões mais importantes foram: confiança nos médicos que cuidam do paciente e ter sempre a mesma equipe médica cuidando de seu familiar. A confiança se mostra importante não só na relação com o paciente, como também para aqueles inseridos no mesmo contexto. O segundo elemento mais importante é interessante, pois, por meio do cuidado de um dado paciente por um mesmo profissional ou equipe ao longo do tempo, há a individualização de seu cuidado.^(9)^ Esse item é listado como relevante não só no nosso estudo^(10,11)^ e, de fato, há uma revisão da literatura sobre a satisfação dos pacientes durante seu tratamento que advoga que, quanto mais personalizado, maiores são seus índices de satisfação.^(9)^ Segundo esse mesmo estudo, a individualização do tratamento faz com que comunicação do paciente com os médicos seja melhor, assim como o envolvimento do paciente com seu tratamento.^(9)^

Já para os pacientes de nosso serviço em cuidados paliativos, o item mais importante foi ter confiança no médico responsável por seu tratamento, item primordial também para os pacientes do estudo canadense.^(4)^ Em um estudo conduzido por Thom et al.,^(12)^ foi correlacionado o comportamento médico com a confiança dos pacientes. Esses autores entrevistaram 414 pacientes que, após consulta, avaliavam seus médicos em relação a 18 comportamentos preestabelecidos, o que permitiu a avaliação do grau de confiança nesses profissionais. Como resultado, os comportamentos mais associados com a confiança no médico foram: demonstrar atenção e cuidado, competência técnica e a qualidade de sua comunicação.

Em relação aos cuidadores de pacientes em cuidados paliativos, os três itens mais importantes lembrados em nosso estudo referiram-se à qualidade da comunicação. No estudo canadense, tal preocupação dos cuidadores apareceu em terceiro lugar, sendo também uma prioridade.^(4)^ No entanto, comunicar-se de forma adequada pode não ser uma tarefa fácil. Estudo de Wilmot e Hocker^(13)^ acerca de médicos e profissionais de saúde que cuidavam de pacientes com câncer em estágio avançado em um centro americano revelou que a parte mais difícil de seus trabalhos era a comunicação com as famílias dos pacientes. Em uma revisão sistemática, em que foram incluídos 21 trabalhos, observou-se correlação positiva entre a efetividade da comunicação entre médico e paciente e o êxito do tratamento. Os aspectos que obtiveram maiores relações com a adequada comunicação foram: controle da dor, aderência ao tratamento, resolução dos sintomas e a saúde emocional.^(14)^

Deste modo, para que possamos melhorar a qualidade de vida dos pacientes e de seus cuidadores, temos que conhecer o que é primordial para ambos os grupos. Percebemos que, para pacientes, ter confiança em seu médico, ser acolhido de forma digna e humanizada e manter-se bem informado sobre sua doença são questões prioritárias. Já para cuidadores, a maior parte dos itens julgados como essenciais refletiu a qualidade da comunicação com os médicos responsáveis pelo cuidado de seus parentes e que estes tivessem adequado controle de seus sintomas. Na trajetória de uma doença crônica e potencialmente letal, como o câncer, temos a oportunidade de perceber quais os valores que permanecem importantes ao longo do tempo. Comparando esses dois estudos em momentos diferentes da evolução natural do câncer em um mesmo serviço de saúde, ficou evidente a importância da confiança na equipe de saúde, tanto ao diagnóstico como nas fases mais avançadas da doença. O conhecimento desses fatores permite melhor focar nossos esforços no que é, de fato, mais importante para pacientes e cuidadores, e esses mesmos itens podem ainda servir como foco de avaliação contínua, para detectar nossas deficiências e progressos nesses mesmos quesitos.^(15)^

## CONCLUSÃO

A confiança mediada por uma boa comunicação e a constância do cuidado são valores tidos como fundamentais para satisfação de cuidadores e de pacientes com câncer durante toda a evolução da doença. A estruturação dos serviços para viabilizar o seguimento longitudinal dos pacientes e o cultivo de habilidades comunicativas na formação médica são, provavelmente, as atitudes mais promissoras para assegurar a satisfação da população de pacientes com câncer e seus cuidadores.
